# Low rate of developing urological complications following kidney transplantation in adults using a stentless ureteral anastomosis

**DOI:** 10.1007/s11255-026-05249-3

**Published:** 2026-07-14

**Authors:** Gaetano Ciancio, Mahmoud Morsi, Adela Mattiazzi, Angel Alvarez, Matthew Gaynor, Jeffrey J. Gaynor

**Affiliations:** 1 Department of Surgery, University of Miami Miller School of Medicine, Jackson Memorial Hospital, Miami Transplant Institute, 1801 NW 9th Ave, 7th Floor, Miami, FL 33136 USA; 2 Department of Urology, University of Miami Miller School of Medicine, Jackson Memorial Hospital, Miami, FL USA; 3 Department of Medicine, Division of Nephrology, University of Miami Miller School of Medicine, Jackson Memorial Hospital, Miami, FL USA; 4 Miami Transplant Institute, University of Miami Miller School of Medicine, Jackson Memorial Hospital, Miami, FL USA

**Keywords:** Kidney transplantation, Stent-less, Urological complications, Urinary leak, Stenosis

## Abstract

**Purpose:**

Severe urologic complications after kidney transplantation may require multiple radiological or surgical interventions.

**Materials and methods:**

We previously reported outcomes using our modified extravesical ureteroneocystostomy technique which was designed to avoid routine stent placement at the time of transplant as well as to reduce post-operative urologic complications. Here, we reviewed 697 adult kidney-alone transplant recipients who were consecutively transplanted by a single surgeon at our center since 2014.

**Results:**

During the first 12mo post-transplant, the observed percentage of patients who developed any urologic complication was 2.3% (16/697), and 2/16 patients developed a 2nd urologic complication (in total, 18 cases). The percentage developing a urinary leak or ureteral obstruction/stricture (including suspected cases) was 2.0% (14/697). Urinary leaks were observed in 9/18 cases, and ureteral obstructions/strictures were observed in 9/18 cases (including 3 UPJ obstructions). Additionally, ureteral necrosis was observed in 3/18 cases. Grade 4 vesicoureteral reflux was observed in 1 case, and one bladder rupture involving the ureteral anastomosis was observed (during an attempted removal of a large, suprapubic hematoma). Half of the 18 urologic cases required surgical intervention, while the rest of them were managed by interventional radiology. These outcomes compare favorably with published series, particularly those using routine ureteral stenting.

**Conclusions:**

Our extravesical ureteroneocystostomy technique appears to significantly reduce early post-transplant urological complications without the need for ureteral stent placement at the time of transplant. However, without having a direct control group, one cannot conclude that our extravesical ureteroneocystostomy approach without stenting is equivalent to optimally performed extravesical Lich-Gregoir ureteroneocystostomies with routine stenting.

## Introduction

Despite advances in surgical techniques in kidney transplantation, urologic complications may still occur in the early post-transplant period, e.g., ureteral obstruction/stricture, either proximally at the ureteropelvic junction (UPJ), mid-ureter, or distally at the ureterovesical junction (UVJ), and urinary leak, which is often accompanied by ureteral necrosis. These complications are associated with a range of potentially unfavorable outcomes, including morbidity, repeat hospitalizations, graft failure, and even mortality [1, 2]. Some urologic complications may require multiple radiological and/or surgical interventions to achieve complete resolution. In defined cohorts of at least 200 patient, the reported incidence of urologic complications following kidney transplantation ranges from 1.7% to 15.0% [1].

The transplanted ureter remains the primary contributor to the development of most of these urologic complications, and it has been argued that to minimize complication risk, a ureteral stent should be routinely placed at the time of transplantation [1–4]. Our transplant program has adopted a standardized ureteral anastomosis technique specifically designed to safely eliminate the routine use of prophylactic ureteral stenting at the time of transplantation as well as to minimize and potentially eliminate the incidence of urologic complications early post-transplant [5].

Included in our original reports [5, 6] of 500 consecutive kidney transplant recipients (who were transplanted by a single surgeon during 2014–2019 and received no stent placement at the time of transplant) were 350 adult, kidney-alone transplant recipients who also received no JP drain placement at the time of transplant. Our more recent report [7] included 657 adults (plus 50 pediatric patients) who received a kidney-alone transplant by the same single surgeon during 2014-February 2023 and who also received no stent placement nor JP drain placement at the time of transplant.

In the current retrospective cohort study, we detail the incidence and spectrum of urological complications among 697 consecutive adult kidney-alone transplant recipients who underwent transplantation between 2014-August, 2023 by the same single surgeon at our center using our standardized ureteral anastomosis technique, without the routine use of prophylactic ureteral stent placement (and also without JP drain placement). While the previous report [7] focused on highlighting 7 surgical modifications to the conventional kidney transplant technique, in this report with 40 additional adult patients we highlight the importance of using our extravesical ureteroneocystostomy technique without ureteral stenting at the time of transplant.

## Materials and methods

### Extravesical ureteroneocystostomy technique without ureteral stenting

A detailed description along with illustrations of our extravesical ureteroneocystostomy technique without ureteral stenting was previously reported in Ciancio et al. [5]. Highlighted in that report was the following, “Four main differences between our extravesical technique and those previously described by Lich-Gregoir [8, 9] and others [10–18] are outlined as follows: i) The bladder is mobilized as much as possible in order to allow it to move upward with the kidney (in case it moves upward) during abdominal closure. Its goal is to provide additional insurance against the risk of any rip or tear in the UVJ anastomosis from occurring, thereby decreasing urinary leak risk; ii) Our ureter has a posterior 2 cm (more wide open) spatulation (thereby lowering ureteral stenosis risk), whereas three other extravesical approaches offer less spatulation, 0.5 cm, 1 cm and 1.5 cm, respectively [13, 16, 17]. During anastomosis of the non-spatulated portion of the ureter to the bladder mucosa, the total circumference of the ureter is included in the running suture, but during anastomosis of the spatulated ureter, only 50% of the ureteral orifice (original and spatulated orifice) or less is incorporated into the suture line. During inflammation and absorption of the absorbable suture, not all of the ureteral orifice is inflamed, thereby possibly further lessening the chance of developing ureteral stenosis [19, 20]; iii) The extravesical ureteral anastomosis is performed with running suture and incorporates both bladder mucosa and some (roughly 30% of) detrusor muscle. The 4 anchor stitches incorporate almost the complete thickness of the bladder wall. This gives added strength to the anastomosis in case of bladder fullness, urinary retention, or mobilization upward of the renal allograft during closure. In recipients having a thin detrusor muscle (i.e., mostly in patients who were transplanted due to polycystic kidney disease or received 8–10 years of dialysis pre-transplant), we were still able to incorporate the detrusor into the anastomosis to help strengthen it (again, further lowering the risk of a urinary leak from occurring); and iv) During suturing of the ureteral medial wall, a right-angle clamp is introduced into the ureteral orifice to ensure that it is not stenosed or incorporated by the running suture, and the final stitch is tested with the right-angle clamp, making sure that it is not too tight to cause stenosis. Use of a right-angle clamp in this fashion was thought to further lower the risk of occurrence of an iatrogenic ureteral stenosis.”

### Data description

Between March 17, 2014 and August 13, 2023, a total of 697 consecutive adult kidney-alone transplant recipients at the Miami Transplant Institute underwent a novel extravesical ureteroneocystostomy performed (by a single surgeon) without ureteral stenting [5]. At our teaching transplant program, kidney transplantation procedures were performed in a collaborative manner, with the attending surgeon completing the initial half portion of the ureteral anastomosis, and the transplant fellow or resident performing the remaining half portion under direct supervision. This retrospective cohort study was approved by the University of Miami Institutional Review Board and adheres to the ethical principles of the Helsinki Declaration, as revised in 2013. All patients (and/or guardians) gave written informed consent to be transplanted; however, study participant consent was waived due to the retrospective nature of the study.

All transplanted deceased donor kidneys had been connected to the LifePort Renal Preservation Machine^®^ prior to transplantation using kidney perfusion solution (KPS-1^®^).

Patients who underwent other reimplantation techniques (performed by other transplant surgeons at our center at the time of transplant) were not included here. All patients in this study had a routine ultrasound of the kidney allograft performed during the first week post-transplant.

Regarding the surgical technique, the allograft was brought into the surgical field, and detailed steps of the surgical approach, including the transplant technique and ureteral anastomosis have been described in previous publications [5, 7].

Clinical outcomes that occurred during the first 12 months post-transplant were analyzed. Since the date of last follow-up was August 30, 2024, all patients were followed for at least 12 months post-transplant.

All urologic complications that developed post-transplant were recorded. Suspected urologic complications were evaluated with renal ultrasonography, MAG-3 (mercaptoacetyltriglycine) scan, and/or antegrade nephrostogram. Urologic complications were treated by either percutaneous radiological procedures, surgery, or a combination of both. Suspected ureteral stenosis was evaluated along with serial monitoring for BK virus replication (in urine and blood).

Voiding cystourethrogram was not routinely performed, as routine evaluation for vesicoureteral reflux was not considered to be necessary in our transplant recipients; therefore, only symptomatic reflux was evaluated and considered as a urologic complication.

### Statistical analysis

The primary outcome variable for this study was the occurrence of any urologic complication during the first 12mo post-transplant. Secondary outcome variables included the (separate) occurrences of distinct types of urologic complications (e.g., urinary leak, ureteral obstruction or stricture, etc.) during the first 12mo post-transplant, delayed graft function, a urinary tract infection (UTI) (or a recurrent UTI) that required hospitalization during the first 12mo post-transplant, and graft loss (death-uncensored) during the first 12mo post-transplant, respectively. Graft loss was defined as the date of graft failure (return to permanent dialysis, graft nephrectomy, or retransplantation) or death, whichever occurred first.

Frequency distributions were determined for categorical baseline and clinical outcome variables. Means and standard errors (SE) (along with medians and ranges) were calculated for continuous baseline and clinical outcome variables. In addition, stepwise Cox regression was performed to determine the significant multivariable predictors (of selected baseline variables) for the hazard rate of developing a urologic complication during the first 12mo post-transplant.

Since there was no control group of patients available from our center in which the more standard extravesical Lich-Gregoir or modified Lich-Gregoir ureteroneocystostomy technique with routine stent placement was used, we performed a descriptive comparison with randomized clinical trials results reported by the meta-analyses of Mangus and Haag [1] and Yin et al. [21] comparing routine vs. no routine stent placement using mainly extravesical ureteroneocystostomies. The primary outcome of interest (as analyzed in each of these clinical trials) was the incidence of any urologic complication, defined as the occurrence of either a urinary leak or a ureteral obstruction/stricture. Attention was paid to ensure that any patients who developed both types of these urologic complications (quite rare as reported in these studies) would be counted only once, not twice.

## Results

### Patient demographics

Distributions of selected baseline variables are presented in Table 1. The percentage of deceased donor recipients was 63.4% (442/697), and a majority of recipients were either black (non-Hispanic), 32.9%% (229/697), or Hispanic, 42.5% (296/697). Mean (± SE) recipient age at transplant was 53.7 ± 0.5 (range: 18.1–82.9) years; mean donor age was 43.4 ± 0.5 (range: 0.2–71.0) years. The percentage of donor kidneys having 1, 2, 3, and 4 donor arteries was 76.0%% (530/697), 20.5% (143/697), 3.3% (23/697), and 0.1% (1/697), respectively. The percentage of patients that had pre-transplant bladder reconstruction was 0.6% (4/697).

**Table 1:** Distributions of selected baseline variables (N = 697)

	Mean ± SE if continuous
Baseline Variable	Percentage with characteristic if categorical
Recipient Age (yr)	53.7 ± 0.5 (N = 697)
	[Median = 55.5, Range: 18.1–82.9]
Recipient Gender:	
Female	35.4% (247/697)
Male	64.6% (450/697)
Recipient Race/Ethnicity	
Black (non-Hispanic)	32.9% (229/697)
Hispanic	42.5% (296/697)
White (non-Hispanic)	22.7% (158/697)
Other	2.0% (14/697)^1^
Recipient BMI (kg/m^2^)	27.4 ± 0.2 (N = 697)
	[Median = 27.0, Range: 16.0–39.5]
Retransplant Status	
Primary	92.8% (647/697)
Retransplant	7.2% (50/697)
Preemptive Transplant	
No	79.3% (553/697)
Yes	20.7% (144/697)
Pretransplant Time on Dialysis (mo)	48.6 ± 5.7 (N = 697)
	[Median = 21.2, Range: 0.0–1410.1]
Cause of ESRD	
ADPKD	9.5% (66/697)
DM	31.0% (216/697)
HTN Only	23.0% (160/697)
FSGS	7.6% (53/697)
IgA Nephropathy	6.0% (42/697)
SLE Only	4.2% (29/697)
Reflux Nephropathy	1.4% (10/697)
Other	17.4% (121/697)
Donor Type	
Living	36.6% (255/697)
Deceased	63.4% (442/697)^2^
Number of Donor Arteries	
1	76.0% (530/697)
2	20.5% (143/697)
3	3.3% (23/697)
4	0.1% (1/697)
Pretransplant Bladder/Urinary Tract Reconstruction	
No	99.4% (693/697)
Yes	0.6% (4/697)
Donor Age (yr)	43.4 ± 0.5 (N = 697)
	[Median = 45.0, Range: 0.2–71.0]
CIT (hr)	20.1 ± 0.6 (N = 697)
	[Median = 23.3, Range: 0.2–69.5]
WIT (min)	27.3 ± 0.3 (N = 697)
	[Median = 26.0, Range: 14.0–94.0]
KDPI (%)	57.7 ± 1.3 (N = 442)
	[Median = 60, Range: 1–99]

^1^Other race/ethnicity included: Asian (13/697) and Native American (1/697)

^2^Among the 442 deceased donor kidneys, 2/442 were pediatric en bloc kidneys

### Urologic complications

Distributions of selected outcome variables are presented in Table 2. Overall, 2.3% of patients (16/697) developed urologic complications. Two patients experienced two separate urological complications each, resulting in a total of 18 urological complications. Twelve patients who experienced urologic complications received a deceased donor kidney; 4 of the patients received a living donor kidney.

**Table 2:** Distributions of selected outcomes variables during the first 12 months post-transplant (N = 697)

	Mean ± SE if continuous
Outcome Variable	Percentage with characteristic if categorical
Developed DGF:	
No	80.6% (562/697)
Yes	19.4% (135/697)
Developed a 1 st Urologic Complication during the First 12mo Post-transplant	
No	97.7% (681/697)
Yes	2.3% (16/697)
Developed a 2nd Urologic Complication during the First 12mo Post-transplant	
No	99.7% (695/697)
Yes	0.3% (2/697)
Developed a 1 st UTI Requiring Hospitalization during the First 12mo Post-transplant	
No	91.2% (636/697)
Yes	8.8% (61/697)
Developed a 2nd UTI Requiring Hospitalization during the First 12mo Post-transplant	
No	97.7% (681/697)
Yes	2.3% (16/697)
Developed (Death Censored) Graft Failure during the First 12mo Post-transplant	
No	98.1% (684/697)
Yes	1.9% (13/697)
Death with a Functioning Graft during the First 12mo Post-transplant	
No	95.7% (667/697)
Yes	4.3% (30/697)
Developed (Death Uncensored) Graft Loss during the First 12mo Post-transplant	
No	93.8% (654/697)
Yes	6.2% (43/697)
eGFR at 1mo post-tx (ml/min/1.73m^2^)^1^	61.9 ± 1.1 (N = 694)
	[Median = 60.1, Range: 4.7–168.9]
eGFR at 6mo post-tx (ml/min/1.73m^2^)^1^	66.3 ± 0.9 (N = 672)
	[Median = 65.2, Range: 6.6–145.4]
eGFR at 12mo post-tx (ml/min/1.73m^2^)^1^	69.1 ± 0.9 (N = 654)
	[Median = 68.2, Range: 6.8–156.1]

^1^The race-based CKD-EPI formula was used to estimate GFR

Among the 16 patients who developed a 1 st urological complication during the first 12 months post-transplant, the types of 1 st urological complications observed were as follows: urinary leak (N = 5: 2 due to necrosis of the ureter at 0.6 and 1.1 months; 1 occurring mid-ureter at 1.3 months; and 2 due to an anastomotic urinary leak at 1.7 and 2.9 months), ureteral obstruction/stricture (N = 7: 3 at the UPJ at 2.3, 4.4, and 10.9 months; 1 at mid-ureter at 4.8 months; 1 at the UVJ with this patient simultaneously experiencing a urinary leak at 0.5 months; 1 at both the UPJ and UVJ due to malrotation of the transplanted kidney at 2.8 months; and 1 throughout the ureter, from the UPJ to UVJ, due to fibrous tissue that completely surrounded the ureter at 1.2 months), distal ureteral necrosis/ischemia without a urinary leak (N = 1: at day 3), urinoma/suspected urinary leak (N = 1: at 2.6 months), Grade 4 vesicoureteral reflux (VUR) (N = 1: at 5.1 months), and bladder rupture/rupture of the ureteral anastomosis (caused by an attempt at removing a large, suprapubic hematoma) (N = 1: at 0.2 months). The latter 2 patients experienced no urinary leak nor ureteral obstruction/stricture. One patient who initially developed an anastomotic urinary leak at 1.7 months subsequently developed an anastomotic ureteral obstruction/stricture at 2.7 months. Another patient who initially developed a mid-ureter urinary leak at 1.3 months subsequently developed a ureteral obstruction/stricture due to fibrous tissue that completely surrounded the ureter (from the UPJ to UVJ) at 5.7 months.

In total, 2.0% (14/697) of the patients developed either a urinary leak, suspected urinary leak, ureteral necrosis/ischemia, and/or a ureteral obstruction/stricture during the first 12mo post-transplant: 1.1% (8/697) of the patients developed a urinary leak, a suspected urinary leak, or had ureteral necrosis/ischemia but without a urinary leak during the first 12mo post-transplant, and 1.3% (9/697) of the patients developed a ureteral obstruction/stricture during the first 12mo post-transplant, with 3/8 and 3/9 of these patients developing both types, respectively.

Treatment of the 2 patients having a urinary leak due to necrosis of the ureter included: debridement of necrotic ureteral tissue, ureteral reimplantation, and nephroureteral stent placement (N = 2). Treatment of the 3 other patients who developed a urinary leak (2 with an anastomotic urinary leak and 1 with a mid-ureteral leak) included IR with nephroureteral stent placement (N = 3). Treatment of the single patient having distal ureteral necrosis/ischemia without a urinary leak included ureteral reimplantation along with nephroureteral stent placement (N = 1). Lastly, treatment of the single patient having a urinoma/suspected urinary leak was IR with nephroureteral stent placement (N = 1).

Treatment of 5 patients who developed a ureteral obstruction/stricture (3 with a UPJ obstruction, 1 at mid-ureter, and 1 at the UVJ who simultaneously experienced a urinary leak) included: IR with ureteral dilatation and nephroureteral stent placement (N = 5). The patient who developed a ureteral obstruction/stricture due to a malrotated transplanted kidney had surgery to place the transplanted kidney in the correct position along with nephroureteral stent placement (N = 1). Treatment of the 2 patients who developed a ureteral obstruction/stricture due to fibrous tissue that completely surrounded the ureter (from the UPJ to UVJ) included: dissection of fibrous tissue and ureteral reimplantation along with nephroureteral stent placement (N = 2). Treatment of the 2nd urological complication for the single patient who developed an anastomotic ureteral obstruction/stricture following an anastomotic ureteral leak included IR with ureteral dilatation and nephroureteral stent placement. Treatment of the single patient having Grade 4 VUR included Deflux^R^ Injection into the ureter via cystoscopy and IR with nephroureteral stent placement (N = 1). Lastly, treatment of the single patient having a bladder rupture/rupture of the ureteral anastomosis included ureteral reimplantation (N = 1).

In total, the percentage of patients who required surgical repair of their original ureteroneocystostomy was 0.9% (6/697) (i.e., 6/16 patients who experienced a urological complication). Including the single patient with an allograft malrotation who required surgical replacement and the single patient with grade 4 VUR who received Deflux^R^ Injection into the ureter via cystoscopy, the total percentage of patients who required any surgical repair of a urological complication was 1.1% (8/697).

Stepwise Cox regression of the hazard rate of developing a urologic complication found that only one baseline characteristic was associated with a significantly higher rate: patients with a pretransplant history of diabetes appeared more likely to develop a urologic complication during the first 12mo post-transplant, 4.2% (9/216), in comparison with patients not having a pretransplant history of diabetes, 1.5% (7/481) (P = 0.02). No significant association of any other baseline factor (such as recipient age, donor age, number of donor arteries, cold ischemia time, and delayed graft function) with the hazard rate of developing a urologic complication was observed (P > 0.05). For instance, the observed percentage of patients developing a urologic complication during the first 12mo post-transplant was 1.9% (10/530) vs. 3.6% (6/167) for recipients of a donor kidney having only 1 vs. ≥ 2 donor arteries (P = 0.20).

### Urinary tract infections and graft loss

The observed percentage of patients who developed a UTI requiring hospitalization during the first 12mo post-transplant was 8.8% (61/697), with 22/61 and 44/61 of these infections occurring during the first month and 3mo post-transplant, respectively. In addition, the observed percentage who developed a recurrent UTI requiring hospitalization during the first 12mo post-transplant was 2.3% (16/697). Of note, while patient numbers were small, the observed incidence of UTI requiring hospitalization during the first 12mo post-transplant which occurred following the development of a urologic complication was 18.8% (3/16), higher in comparison with UTI incidence occurring among those not developing a urologic complication (8.1%; 55/681) (P = 0.02, Cox model time-dependent covariate likelihood ratio test) (note: in 3/16 patients who developed a urologic complication, a UTI requiring hospitalization occurred prior to the development of the urologic complication).

The overall percentage of patients who developed (death uncensored) graft loss during the first 12mo post-transplant was 6.2% (43/697), with 1.9% (13/697) experiencing death-censored graft failure, and 4.3% (30/697) experiencing death with a functioning graft. Of note, none of the 16 patients who developed a urologic complication subsequently developed death-censored graft failure during the first 12mo post-transplant. A single patient who developed a distal ureteral ischemia/necrosis without a leak at day 3 post-transplant subsequently died with a functioning graft due to an acute pulmonary embolism at 2.9mo post-transplant. Thus, the overall percentage of patients who developed graft loss during the first 12mo post-transplant was quite similar between those who developed a urologic complication, 6.3% (1/16), and those who did not develop a urologic complication, 6.2% (42/681).

### Comparing our percentage who developed a urologic complication (Urinary Leak or Ureteral Obstruction/Stricture) with those of 10 randomized trials using extravesical ureteroneocystostomies and routine stenting

Percentages of patients developing a urologic complication post-transplant, as reported by 10 randomized clinical trials of routine vs. no routine stent placement in kidney transplantation using extravesical ureteroneocystostomies, appear in Table 3 [22–31]. Of note, 6 of the randomized clinical trials reported by Yin et al. [21] were not utilized here for the following reasons: ureteral obstructions/strictures were not reported in 2 studies (only clinically observed urinary leaks were reported), presentation of results was not sufficiently clear in 1 study, and 3 reports were unretrievable both by us and our university library. Combining the routinely stented groups across these 10 randomized trials, the overall percentage of patients who developed a urologic complication was 2.5% (21/857), slightly higher than our overall percentage of 2.0% (14/697).

**Table 3:** Percentage Developing a Urologic Complication (Urinary Leak or Ureteral Obstruction/Stricture), as Reported by 10 Randomized Clinical Trials of Routine vs. No Routine Stent Placement in Kidney Transplantation Using Extravesical Ureteroneocystostomies

	Post-transplant		
	Patient	Percentage Developing a Urologic Complication	
Study Description	Follow-up	Routine Stent Placed	No Routine Stent Placed
Pleass et al. (1995) [22]:	3mo	0.0% (0/75)	13.3% (10/75)
Basseri et al. (1995) [23]:	7–17mo	0.0% (0/35)	8.1% (3/37)
Benoit et al. (1996) [24]:	Mean: 2 yr	1.0% (1/97)	8.2% (8/97)
Kumar et al. (1998) [25]:	12mo	0.0% (0/57)	7.0% (3/43)
Dominguez et al. (2000) [26]:	3mo	3.5% (5/143)	6.6% (9/137)
Osman et al. (2005) [27]:	7–16mo	4.0% (2/48)	0.0% (0/50)
Tavakoki et al. (2007) [28]:	3mo	0.9% (1/112)	16.7% (13/78)
Zaki et al. (2013) [29]:	Mean: 2.1 yr	2.0% (3/150)	2.7% (4/150)
Ooms et al. (2020) [30]:	12mo	8.0% (8/100)	24.0% (24/100)
Vinayak et al. (2025) [31]:	3mo	2.5% (1/40)	5.0% (2/40)
Combined:		2.5% (21/857)	9.4% (76/807)

The standard or modified extravesical Lich-Gregoir ureteroneocystostomy technique was used for all patients in these studies with one exception: as stated in Benoit et al. [24], “A Lich-Gregoir anastomosis was used when the bladder permitted (180 out of 194 cases), but when bladder capacity was too low of if there was a thick detrusor, a Leadbetter procedure was performed.”

After careful evaluation of each study shown in this table, only the report by Benoit et al. [24] stated that 2 patients in the non-stented group developed both a urinary leak and a ureteral obstruction/stricture (thus, a total of 10 urological complications were observed in that group. In no other study was it reported that any patients developed both a urinary leak and a ureteral obstruction/stricture

One patient in the stent group of the Zaki et al. study [29] was reported to have developed ureteric necrosis (but without an apparent urinary leak). In keeping with how we reported our results, this event was included as a urologic complication

Six other randomized clinical trials as reported by Yin et al. [21] were not utilized here for the following reasons: ureteral obstructions/strictures were not reported in 2 studies (only clinically observed urinary leaks were reported), presentation of results was not sufficiently clear in 1 study, and 3 reports were unretrievable both by us and our university library

## Discussion

Kidney transplantation has evolved over decades to become the most effective treatment for end stage kidney disease (ESKD), offering the best long-term patient survival, improved quality of life, and greater cost-effectiveness compared to dialysis [32]. However, the risk of developing urologic complications (especially in the early post-transplant period) remains a significant concern due to its potential impact on patient morbidity and graft survival. To address this issue, the prophylactic use of ureteral stents at the time of kidney transplantation has been shown to significantly reduce the incidence of major urological complications such as ureteral leakage and stricture compared with no stenting, making it a widely adopted preventive measure [1, 21, 33]. Placement of a routine ureteral stent during kidney transplantation has also been the focus of our research, as we have explored the development of a ureteral anastomosis technique that avoids routine ureteral stent placement at the time of transplant [5].

Mangus and Haag [1] in their meta-analysis previously demonstrated a significant difference in the post-transplant incidence of urologic complications seen in 5 randomized, controlled studies of “routine” vs. “no routine” ureteral stent placement at the time of kidney transplant and extravesical (Lich-Gregoir) ureteroneocystostomy: 1.5% (6/407) vs. 9.0% (35/389). In a more recent meta-analysis by Yin et al. [21] that pooled data across 16 randomized controlled trials involving 2486 kidney transplant recipients and compared clinical outcomes with and without prophylactic ureteral stent placement during ureteroneocystostomy, a lower incidence rate of urine leakage was demonstrated in the stent group compared to the no-stent group (RR = 0.25, 95% CI [0.13–0.47]). Ureteral obstruction or stricture was reported in 14 of these studies including 2336 patients, and their meta-analysis also revealed a reduced incidence of this complication in the stent group (RR = 0.42, 95% CI [0.25–0.71]) [21].

Combining the results across 10 randomized trials reported in the meta-analyses of Mangus and Haag [1] and Yin et al. [21] in which extravesical ureteroneocystostomies and routine stenting were used, we showed that the overall percentage of patients who developed a urologic complication (combining either urinary leak or ureteric obstruction/stricture) post-transplant was 2.5% (21/857), slightly higher than our overall percentage of 2.0% (14/697). Furthermore, if one considers the overall percentage of patients who developed a urologic complication among 44 nonrandomized cohort studies reported by Mangus and Haag [1] in which extravesical ureteroneocystostomies and routine stenting were used, inclusion of their observed incidence of 3.2% (137/4245) into an overall calculation would clearly increase the overall percentage. Similarly, in a large retrospective review by Whang et al. [4], in which a single transplant surgeon performed all Lich-Gregoir ureteroneocystostomies with routine stenting, the overall incidence of urologic complications (excluding late complications) was 2.5% (95/3856). In summary, we believe that our results using a modified extravesical ureteroneocystostomy technique but without stenting at the time of transplant matches the best results reported by these other studies in which Lich-Gregoir extravesical ureteroneocystostomies and routine stenting at the time of transplantation were used.

Our overall incidence of any urologic complication developing was 2.3% (16/697 patients), with a total number of 18 urologic complications being recorded among these 697 patients during the first 12mo post-transplant, as two patients each experienced two distinct urologic complications. In addition, the total percentage of patients who required any surgical repair of a urological complication was 1.1% (8/697). Thus, we believe that our low urological complication rate with a stent-less technique is at least comparable to, and within the range of outcomes reported in these other series on utilizing routine ureteral stenting.

An additional advantage in performing stent-less ureteral anastomosis was that none of our patients required an extra surgical procedure simply for stent removal (except for those who required stent placement as treatment for the occurrence of a post-transplant urologic complication). Another important consideration regarding the use of ureteral stent is the incidence of urinary tract infections (UTIs). In the recent report by Yin et al. [21], the incidence of UTI was higher in the stented group, 23.2%, compared to 16.6% in non-stented group, yielding a statistically significant difference. Lastly, while relatively rare, stent migration can occur in routinely stented patients, which requires prompt removal to avoid patient morbidity.

UTI is a common complication after kidney transplantation which accounts for approximately 45% to 72% of all infections occurring during the first 3mo post-transplant [34]. Abbott et al. reported a cumulative UTI incidence of 17% during the first 6mo post-transplant [35]. Our observed incidence of UTI requiring hospitalization during the first 12mo post-transplant was 8.8% (61/697). Additionally, with only 2.3% (16/697) of our cohort developing a recurrent UTI during the first 12mo post-transplant, these low UTI rates could be related (at least in part) to the absence of routine ureteral stent placement and early Foley catheter removal (by 4–5 days post-transplant) [5–7].

Vesicoureteral reflux (VUR) is another complication of the ureteral anastomosis that can occur following kidney transplantation [36], and creation of an anti-reflux tunnel [37] at the time of the ureteral anastomosis can prevent its occurrence [38]. In our technique, an anti-reflux tunnel is created by incorporating additional detrusor layers to prevent vesicoureteral reflux. At our center, VUR is only investigated at our center if the patient becomes symptomatic and develops multiple UTIs. Symptomatic VUR was observed in only 1/697 of our patients, secondary to poor bladder compliance, and required surgical intervention to resolve the complication.

Since urinary leakage and particularly ureteral necrosis can be triggered by compromised ureteral blood flow, a technical aspect of the ureteral anastomosis is the preservation of periureteral tissue [39]. We observed 3 patients who developed ureteral necrosis, despite the fact their ureters had intact periureteral fat with periureteral vessels [40]. Some of the other transplanted ureters did not have intact periureteral fat with periureteral vessels, but ureteral necrosis was not observed in those cases. Thus, while the presence of an intact ureteral wall blood flow is important in avoiding the occurrence of ureteral necrosis [41], it may still occur despite the presence of good ureteral anastomosis with good periureteral tissue preservation. Another factor related to ureteral blood flow and possibly an increased risk of developing a post-transplant urologic complication is the presence of multiple renal arteries in the donor kidney [39, 42]. While 24.0% (167/697) of our kidney transplant recipients received a donor kidney with more than one renal artery, its presence was not predictive of the risk of developing a post-transplant urologic complication.

Furthermore, we identified 5 patients with UPJ obstruction (N = 3) and combined UPJ and UVJ obstruction associated with extensive fibrotic tissue involvement of the entire ureter (N = 2). This represents a highly uncommon urological complication that is rarely described in the literature [21]. Whang et al. [4] reported the incidence of de novo UPJ obstruction in 10 patients out of a series of 4000 kidney transplant recipients (0.25%); the majority of these cases were probably managed by interventional radiology [4]. While we only have documentation that 2/5 of our UPJ obstruction cases were caused by fibrotic scarring, we are concerned that fibrotic scarring may actually be associated with most of the UPJ obstruction cases. In the single additional case of de novo UPJ obstruction in our cohort, which occurred secondary to graft malrotation, surgical repositioning of the transplanted kidney was required.

A major study limitation is the fact that we had no direct control group, i.e., a routine ureteral stenting cohort. Clearly, the results reported here were not based on a randomized controlled study, nor was there an available control group of transplanted patients at our center who received one of the more conventional extravesical ureteroneocystostomies. Second, as routine postoperative assessment for VUR was not performed, the true incidence of asymptomatic reflux may be underestimated. Again, an exact determination as to whether our modified extravesical ureteroneocystostomy approach to create an anti-reflux tunnel by incorporating additional detrusor layers prevents vesicoureteral reflux occurrence would require a direct control group for comparison. Third, given the low number of urologic complication events (n = 16), our multivariable Cox regression analysis may be limited by an insufficient “event-per-variable” ratio, potentially affecting the robustness of the findings. Thus, the observed significant association of having pretransplant diabetes with a higher incidence rate of developing urologic complications should be verified first either by another center’s study or by longer follow-up in our cohort prior to giving it more serious consideration. Fourth, since our UTI definition included only those urinary tract infections that required hospitalization, the true incidence of post-transplant UTIs may have been underestimated in our cohort. Lastly, follow-up in this study was limited to the first 12mo post-transplant; thus, a more accurate assessment of the true incidence of urologic complications in our cohort will likely require longer post-transplant follow-up.

## Conclusion

Our overall incidence of urological complications was 2.3% (16/697) of the patients. A total of 18 urologic complications were recorded, as two patients experienced two separate complications each. In total, 2.0% (14/697) of the patients developed either a urinary leak, suspected urinary leak, ureteral necrosis/ischemia, and/or a ureteral obstruction/stricture during the first 12mo post-transplant. Therefore, our incidence of urologic complications using a stent-less ureteral anastomosis compares favorably with published reports, particularly in previously reported randomized trials evaluating Lich-Gregoir ureteroneocystostomies with routine stenting. Our extravesical ureteroneocystostomy technique appears to significantly reduce early post-transplant urological complications without the need for ureteral stent placement at the time of transplant. However, without having a direct control group, one cannot conclude that our extravesical ureteroneocystostomy approach without stenting is equivalent to optimally performed extravesical Lich-Gregoir ureteroneocystostomies with routine stenting.

## Data Availability

De-identified research data will be made available upon reasonable request.
